# DNA Double Strand Breaks Occur Independent of AID in Hypermutating Ig Genes

**DOI:** 10.1080/10446670310001626571

**Published:** 2003

**Authors:** Linda Bross, Heinz Jacobs

**Affiliations:** Department Immunology University of Maastricht Research Institute Growth and Development Universiteits Singel 50 Maastricht 6200 MD The Netherlands

## Abstract

Somatic hypermutation (SHM) and class switch recombination (CSR) take place in B cells of the germinal center (GC) and are associated with DNA double-strand breaks (DNA-DSBs). Transcription favors the generation of DNA-DSBs in the V-regions and switch regions of Ig genes. Both SHM and CSR are controlled by the Activation Induced Cytidine Deaminase (AID), an enzyme exclusively expressed in B cells of the GC. Because AID is capable of deaminating deoxy-cytidine (dC) to deoxy-uracil (dU), it might directly induce nicks (single strand DNA breaks) and also DNA-DSBs via a U-DNA glycosylase mediated base excision repair pathway ('DNA-substrate model'). Alternatively, AID could function like its closest homologue Apobec-1 as a catalytic subunit of a RNA editing holoenzyme ('RNA-substrate model'). To determine whether AID lies upstream or downstream of the DNA lesions found in hypermutating Ig genes, we have analysed the Vλ locus of AID proficient and AID deficient GC B cells for the presence of DNA-DSBs. Although rearranged Vλ genes are preferred targets of SHM we find that AID-proficient and -deficient Vλ1/2-expressing GC B cells display a similar frequency, distribution and sequence preference of DNA-DSBs in rearranged and germline Vλ genes, favoring the idea that AID acts downstream of the DNA lesions to mediate error prone processing.


*Clinical & Developmental Immunology*, June–December 2003 Vol. 10 (2–4), pp. 83–89

Taylor & Francis healthsciences

### DNA Double Strand Breaks Occur Independent of AID in Hypermutating Ig Genes

LINDA BROSS and HEINZ JACOBS*

*Department Immunology, University of Maastricht, Research Institute Growth and Development, Universiteits Singel 50, 6200 MD Maastricht, The Netherlands*

Somatic hypermutation (SHM) and class switch recombination (CSR) take place in B cells of the germinal center (GC) and are associated with DNA double-strand breaks (DNA-DSBs). Transcription favors the generation of DNA-DSBs in the V-regions and switch regions of Ig genes. Both SHM and CSR are controlled by the Activation Induced Cytidine Deaminase (AID), an enzyme exclusively expressed in B cells of the GC. Because AID is capable of deaminating deoxy-cytidine (dC) to deoxy-uracil (dU), it might directly induce nicks (single strand DNA breaks) and also DNA-DSBs via a U-DNA glycosylase mediated base excision repair pathway ('DNA-substrate model'). Alternatively, AID could function like its closest homologue Apobec-1 as a catalytic subunit of a RNA editing holoenzyme ('RNA-substrate model'). To determine whether AID lies upstream or downstream of the DNA lesions found in hypermutating Ig genes, we have analysed the Vλ locus of AID proficient and AID deficient GC B cells for the presence of DNA-DSBs. Although rearranged Vλ genes are preferred targets of SHM we find that AID-proficient and -deficient Vλ1/2-expressing GC B cells display a similar frequency, distribution and sequence preference of DNA-DSBs in rearranged and germline Vλ genes, favoring the idea that AID acts downstream of the DNA lesions to mediate error prone processing.

*Keywords*: Activation induced cytidine deaminase (AID); Class switch recombination (CSR); DNA Double-Strand Break (DSB); Immunoglobulin lambda (Igλ); Somatic hypermutation (SHM)

### INTRODUCTION

Somatic hypermutation (SHM) of B cell Immunoglobulin (Ig) variable region genes and class switch recombination (CSR) in the Ig constant region genes allow further diversification of the primary antibody repertoire and increase the efficacy of the immune response. SHM and CSR are processes that: (a) take place in B cells of the GC (Neuberger and Milstein, 1995) (b) require the activation induced cytidine deaminase (AID) (Muramatsu *et al.*, 2000) (c) are associated with DNA lesions (single strand and double-stranded breaks) (Bross *et al.*, 2000; Papavasiliou and Schatz, 2000) (Kong and Maizels, 2001) and (d) are dependent on the transcription of the Ig-V region (Jacobs *et al.*, 1999) and the -switch regions (Honjo *et al.*, 2002), respectively. While point mutations and small deletions are found in the V (Goossens *et al.*, 1998) as well as switch regions (Petersen *et al.*, 2001; Nagaoka *et al.*, 2002), the termination of a CSR involves deletion of a large fragment of intervening DNA between two active switch regions. In contrast to SHM, CSR involves the DNA dependent protein kinase (DNA PKcs) an enzyme involved in non-homologous end-joining (Bemark *et al.*, 2000) (for review, see also Honjo *et al.*, 2002). Thus while previously the molecular mechanisms leading to CSR and SHM appeared to be distinct, the above mentioned parallels (see also Table I) favor a common mechanism in the initiation of CSR and SHM.

Mutations in the GC specific enzyme AID are causative for the autosomal recessive form of the human hyper Ig M syndrom 2 (HIGM2)(Revy *et al.*, 2000). AID controls not only SHM and CSR (Muramatsu *et al.*, 2000) but, as was shown recently in DT40 B cells, also gene conversion in avian Ig genes (Arakawa *et al.*, 2002). As AID can mediate CSR of a recombination substrate in fibroblasts (Okazaki *et al.*, 2002) and hybridomas (Martin *et al.*, 2002), AID seems to be the only GC specific factor required for the induction of SHM and CSR. The close homology of AID to the RNA editing enzyme APOBEC-1 (Muramatsu *et al.*, 1999) suggests that AID might be involved in RNA editing. Given this homology and the fact that APOBEC-1 requires the Apobec-1 Complementation Factor (ACF) (Lellek *et al.*, 2000; Mehta *et al.*, 2000) to deaminate $C^{6666}$ of the apolipoprotein B mRNA to Uracil, AID might also function together with an ACF-like factor in RNA-editing. However, as shown *in vitro*,

*Corresponding author. Tel.: +31-0-43-38-8-2114. Fax: +31-0-43-38-8-4164. E-mail: h.jacobs@immuno.unimaas.nl

ISSN 1740-2522 print/ISSN 1740-2530 online © 2003 Taylor & Francis Ltd
DOI: 10.1080/10446670310001626571

TABLE I Comparing the features of SHM and CSR. See reviews: Jacobs and Bross (2001) and Honjo *et al.* (2002)

| | SHM | CSR |
|---|---|---|
| AID | Required | Required |
| DNA strand breaks | Occur | Occur |
| Transcription | Dependent | Dependent |
| Point mutations | Yes | Yes |
| Small deletions | Yes | Yes |
| Deletion of large DNA regions | No | Between two active switch regions |
| Non-homologous end joining | Independent | Generally required to terminate the deletion process |

AID deaminates deoxy-cytidine (Muramatsu *et al.*, 1999), implicating two principle models of AID to function, a "RNA substrate model" and a "DNA substrate model", respectively. In the "DNA substrate model", AID acts within a nucleoprotein complex to deaminate deoxy-cytidines within ssDNA of transcribed V and switch regions. As shown in Fig. 1a, the presence of deoxy-uracil leads to a recruitment of the base excision repair machinery, causing nicks and/or staggered breaks which might become processed in an error prone fashion. This model suggests that DNA-DSBs normally associated with SHM of Ig V region genes are a direct product of AID action. Alternatively, AID might function as its closest homologue APOBEC-1 by editing a mRNA encoding a SHM/CSR control factor. This control factor might either induce the generation of DNA-DSBs or be involved at a post-cleavage repair step of the DNA-DSBs associated with SHM and CSR (Fig. 1b). Given the absolute requirement of AID in SHM, CSR and gene conversion, knowledge of AID function will provide the key information to understand how GC B cells are enabled, to increase the affinity of the antigen specific Ig pool, change the effector/homing function by isotype switching, and diversify the avian Ig repertoire by gene conversion. Identifying the system that changes the mutation rate from $10^{-9}$ (spontaneous mutation rate) to about $10^{-3}$ base pairs per generation in a higher eukaryotic cell is the focus of SHM research. This manuscript summarizes our findings on the role of transcription and the occurrence as well as the distribution of DNA-DSBs in hypermutating Ig genes of AID proficient and AID deficient GC B cells (Fukita *et al.*, 1998; Bross *et al.*, 2000).

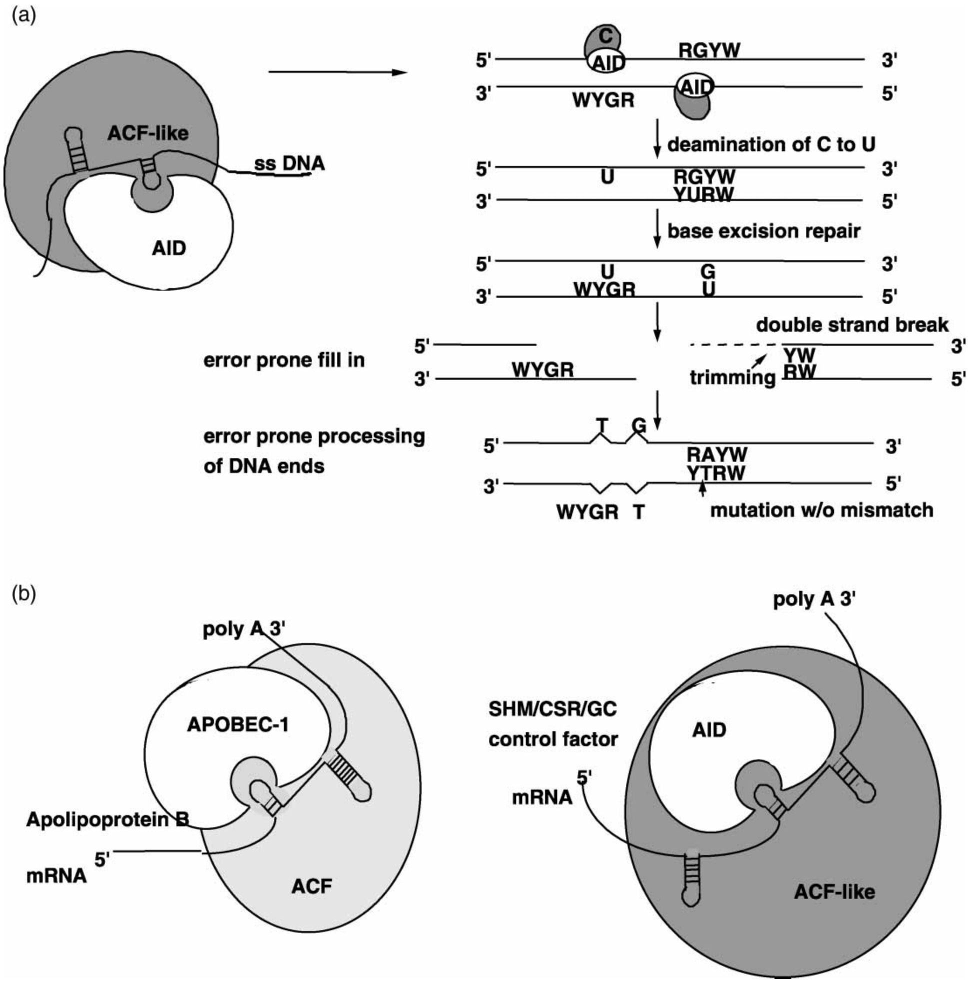

FIGURE 1 (a) "DNA-Substrate Model" (see text) (Jacobs and Bross, 2001). (b) "RNA-Substrate Model" (see text) (Jacobs and Bross, 2001).

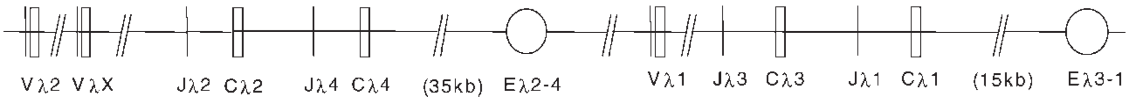

FIGURE 2 Genomic organization of the mouse Igλ locus and λ LC usage (see text).

### RESULTS AND DISCUSSION

We and others have previously demonstrated the frequent occurrence of DNA-DSBs or nicks in hypermutating Ig genes (Jacobs and Bross, 2001). The introduction of DNA-DSBs is favored by transcription and consistent with the mutations in hypermutated V region genes, about 60% were found in RGYW/WRCY (R = A or G, Y = C or T, W = A or T). The RGYW motif and its inverse complement WRCY are hot spots intrinsic to the hypermutation mechanism. While these data clearly demonstrated the presence of breaks in rearranged Ig V regions, the situation of non-rearranged i.e. germline (gl) V genes in GC B cells has not been addressed so far. This question is particularly interesting in view of the fact that normally rearranged V genes mutate at a higher frequency as germline (gl) V genes (Azuma, 1998). To address this issue, we have made use of Ig kappa (κ) deficient mice, where only B cells expressing a functionally rearranged Ig lambda (λ) light chain (LC) can develop (Zou *et al.*, 1993). A schematic outline of the mouse Igλ LC locus is shown in Fig. 2. The murine Igλ LC locus likely arose by gene duplication and comprises two functional V genes (Vλ1 and Vλ2) and a pseudogene (VλX). Vλ1 is preferentially rearranged to Jλ3 and less frequently to Jλ1 and more than 90% of Igλ expressing B cells make use of Vλ1 rather than Vλ2. Vλ2 is rearranged preferentially to Jλ2 and less frequently to Jλ4. The duplication of the λ locus also included the enhancer giving rise to two independently active λ enhancers (Eλ2-4 and Eλ3-1). While one enhancer activates one promotor at a given timepoint, two autonomous enhancers should activate two promotors e.g. the promotor of a rearranged as well as a non-rearranged Vλ1 gene as present in most Igλ positive B cells. This situation provides an ideal system to investigate the occurrence, frequency and distribution of DNA-DSBs in germline versus rearranged Vλ genes of GC B cells. Therefore, AID proficient, κ deficient mice and AID deficient mice were immunized. Ten days later GC B cells ($PNA^{high}$, $CD19^{+}$, Propidium $Iodide^{-}$ ($PI^{-}$),

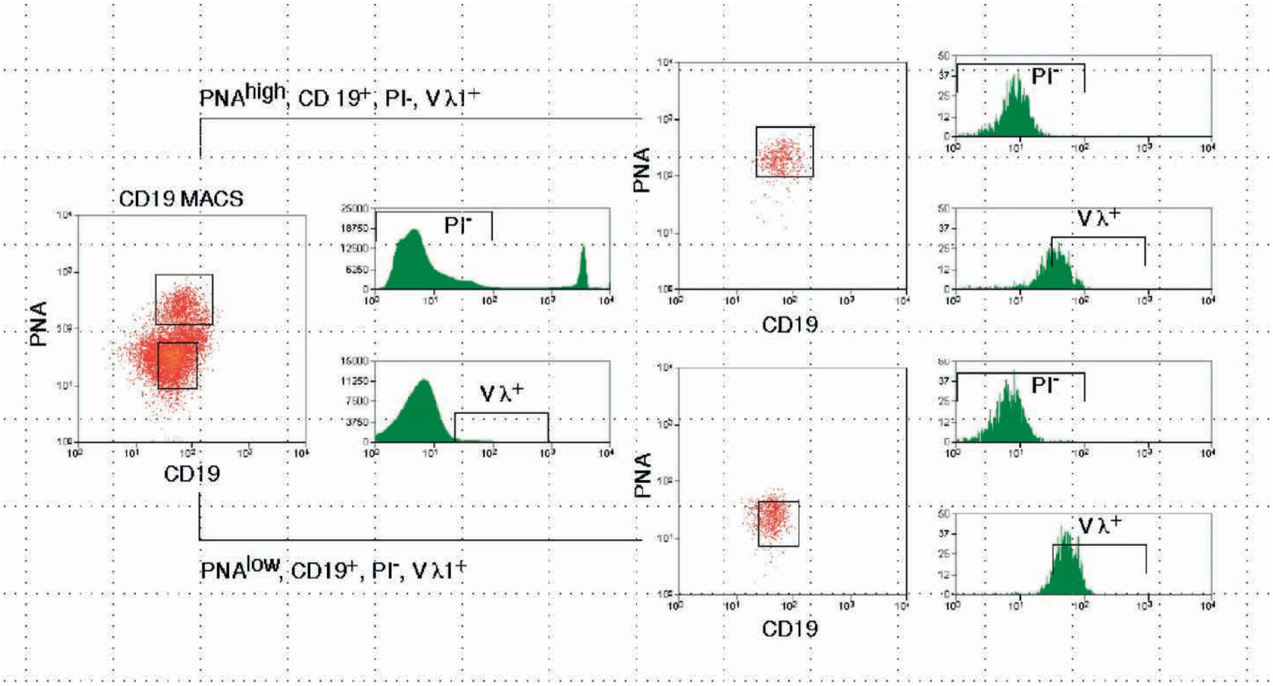

FIGURE 3 Sorting of Vλ1/$2^{+}$ GC and non-GC B cells: Splenic B cells were positively enriched by magnet activated cell sorting (MACS) using CD19-specific beads. AID-deficient B cells are shown as an example. Hereafter cells were stained with a FITC-conjugated CD19 specific monoclonal antibody (clone 1D3), a PE-conjugated Vλ1/2 specific monclonal antibody (clone LS136), and biotinylated peanut agglutinin (PNA). Biotinylated PNA was revealed indirectly with Streptavidin–Allophycocyanin (SA–APC, Molecular Probes). GC-B cells ($PNA^{high}$) in which SHM is known to be ongoing have compared to non-GC B cells ($PNA^{low}$) more binding sites for peanut agglutinin (PNA). Dead cells were excluded by propidium iodine staining ($PI^{+}$). Viable ($PI^{-}$) B cells ($CD19^{+}$) were sorted into a $PNA^{low}$, Vλ1&$2^{+}$ and a $PNA^{high}$, Vλ1&$2^{+}$ fraction (Moflo® Cytomation).

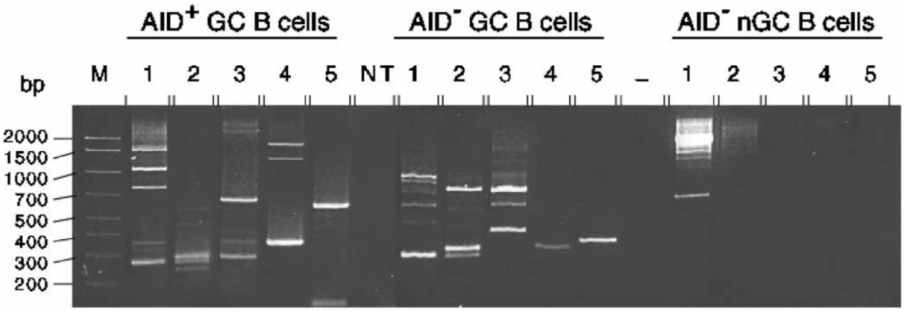

FIGURE 4 DNA-DSBs in the Igλ locus of GC B cells derived from AID proficient, Igκ k.o. mice (AID$^{+/+}$) and AID deficient mice (AID$^{-/-}$). (a) Detection of DNA-DSBs. LM-PCR products of the 5′ break sites from 10000 (1), 5000 (2), 2500 (3), 1250 (4) and 625 (5) cell-equivalents of GC B cells (CD19$^{+}$, PI$^{-}$, PNA$^{high}$) and non-GC B cells (CD19$^{+}$, PI$^{-}$, PNA$^{low}$). LM-PCR products were separated on a 2% (w/v) TAE agarose gel and visualized with ethidium bromide under UV-light. The DNA size markers are indicated in base pairs. As revealed by Southern-blotting and sequencing, most PCR products are Vλ1&2 specific.

Vλ1/2$^{+}$) and non GC B cells (PNA$^{low}$, CD19$^{+}$, PI$^{-}$ and Vλ1/2$^{+}$) were sorted from the spleens of these mice using a highspeed cell sorter (an example is shown in Fig. 3). From the sorted AID proficient and AID deficient B cell fractions, high molecular weight DNA was isolated and aliquots corresponding to a defined number of cells were ligated to blunt ended DNA adapters (splinkerettes) (Bross *et al.*, 2000). The Vλ/adapter hybrids were PCR amplified in two rounds. The two primer sets anneal specifically within the 5′ region of Vλ1&2 genes and to the complement of the long strand of the splinkerette, respectively (Bross *et al.*, 2000). If DNA-DSBs exist in the Vλ1&2 region of hypermutating B cells, the PCR products should occur within the hypermutation domain. The 5′ boundary of the hypermutation domain lies downstream of the promoter transcription initiation site, increases rapidly along the leader and V(D)J exon and decreases beyond the J region. As previously found in targeted V$_H$B1-8 genes (Bross *et al.*, 2000), distinct Vλ specific PCR products were derived from hypermutation competent GC B cells and only infrequently in small, non GC B cells (Fig. 4) (Bross *et al.*, 2002). Southern-blot analysis with a radiolabeled Vλ probe and sequencing revealed the specificity of nearly all PCR products (data not shown). The identity, location and site preference of DNA-DSBs in the Igλ locus were determined by sequencing the cloned PCR products. DNA-DSBs are found in a region of 100–2000 base pairs downstream of rearranged Vλ1&2 genes and interestingly at a similar frequency in non-rearranged, i.e. germline configured Vλ1&2 segments of the λ LC locus (Fig. 5a+b). Fifty-seven percent (43/76) of all DNA-DSBs lie within a RGYW/WRCY motif even though only 38% of randomly distributed DNA-DSBs are expected to occur at these sites, indicating a preference of DNA-DSBs to occur in RGYW/WRCY motif. A web supplement showing the exact location of the breaks has been published elsewhere (Bross *et al.*, 2002).

The presence of a Jλ element or a recombination signal sequence (RSS) at the 3′ end of Vλ segments allows to distinguish between DNA-DSBs in rearranged versus germline Vλ genes. Excluding the DNA-DSBs within Vλ segments, the relative frequency of DNA-DSBs in rearranged versus germline Igλ gene segments can be determined. Of the remaining 29 DNA-DSBs, 48% (14/29) of the DNA-DSBs were found downstream of rearranged and 52% (15/29) downstream of germline Vλ1 gene segments. As transcription favors the generation of DNA-DSBs and each of the two autonomous Igλ enhancers, Eλ2-4 and Eλ3-1 can independently activate transcription of rearranged and non-rearranged Vλ genes, a high frequency of DNA-DSBs in germline Vλ gene segments is expected. Regarding DNA-DSBs in Vλ2 gene segments, only 14% (2/14) are found downstream of rearranged and 86% (12/14) downstream of germline Vλ2 gene segments. This finding likely relates to the fact that VJ rearrangements at the Igλ locus of B cell precursors preferentially ($>90\%$) make use of Vλ1 segments, leaving most Vλ2 alleles ($>90\%$) in germline configuration. In this context it should be mentioned, that according to the enhancer flip flop model (Wijgerde *et al.*, 1995) a single enhancer suffices to activate sequentially several promoters. Therefore, DNA-DSBs in germline V$_H$ or Vk gene segments are also expected to be introduced, albeit taking the cooperation between the intronic and 3′ enhancers at the IgHC and Igk LC locus and the distance to upstream V gene promoters into account at lower frequency.

Comparing AID proficient and AID deficient GC B cells no obvious qualitative nor quantitative differences with regard to DNA-DSBs were found. Thus, regarding the majority of DNA-DSBs, AID deficiency appears not to affect the generation, frequency (number of PCR products) and distribution of DNA-DSBs (size range of PCR products) along the rearranged and germline Vλ1&2 genes of GC B cells in our assay (Figs. 4, 5b). Again, considering the 19 tetramers with a RGYW consensus in Vλ1&2 segments, 38% of randomly distributed DNA-DSBs are expected to occur at these sites, 56% (15/27) of all DNA-DSBs found in AID-deficient GC B cells locate at a RGYW consensus motif. As summarized in Figs. 4

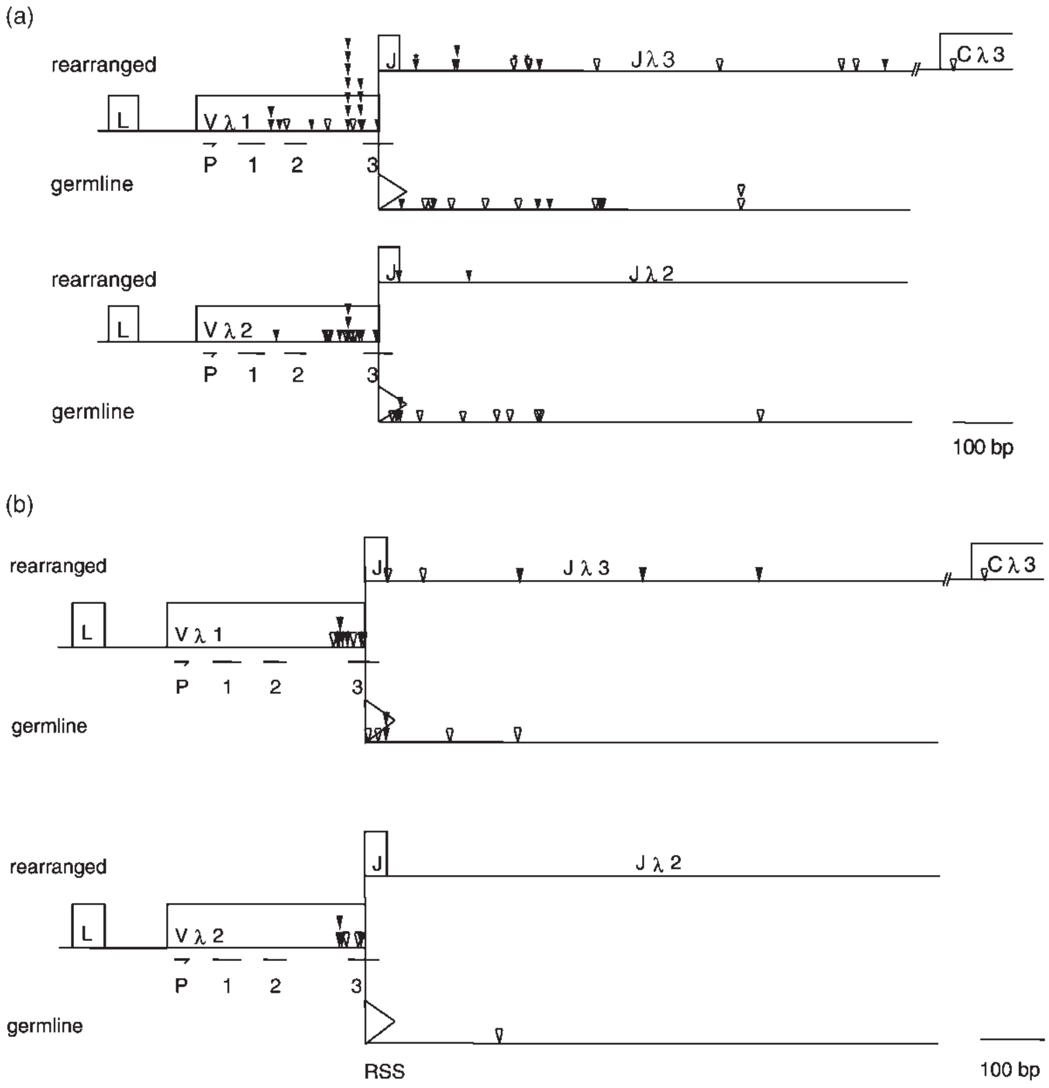

FIGURE 5 (a) DSBs in rearranged and germline Vλ gene segments of hypermutating Igκ deficient GC B cells. The distribution and location of the DSBs found are depicted as arrows. Vertically aligned arrows indicate DSBs obtained from independent LM-PCR reactions. Closed arrows indicate DNA-DSBs at an RGYW motif, open arrows indicate DNA-DSBs outside an RGYW motif. P = location of the Vλ internal primer. 1, 2, 3 = location of the complementary determining regions (CDR1, 2 and 3 respectively). Asterisks above arrows indicate DSBs in Vλ1Jλ3 rearranged λ light chain genes. Triangles indicate the RSS. (b) DSBs in the Igλ locus of AID deficient GC B cells and non-GC B cells. (i) AID does not control the generation of DSBs in hypermutating Ig genes derived from the amplification of the 5′ break sites in Vλ1&2 gene segments from 10000 (1), 5000 (2), 2500 (3), 1250 (4) and 625 (5) cell-equivalents of GC B cells (Vλ +, $CD19^{+}$, $PI^{-}$, $PNA^{high}$) from AID $^{+/+}$ and AID $^{-/-}$ mice as well as non-GC B cells (Vλ +, $CD19^{+}$, $PI^{-}$, $PNA^{low}$) from AID deficient mice (ii) DSBs in rearranged and non-rearranged Vλ genes of GC B cells from AID deficient mice (see text). For annotations see 5(a).

and 5b, the frequency, distribution as well as the preference of DNA-DSBs to occur in RGYW motifs appear not to be controlled by AID (The exact location of the breaks can be found as a web supplement within Bross *et al.* (2002)).

If rearranged and germline-configured, Vλ genes are equal substrates of an unknown nuclease, and are also equally targeted by the SHM system? To (re)-address this question in our system the mutation frequencies of rearranged and non-rearranged Vλ1 genes were determined by amplifying and sequencing these regions from single class-switched $V\lambda1^{+}$, $CD19^{+}$, Igμδ memory B cells isolated from the spleen of C57Bl/6 mice (Bross *et al.*, 2002). Despite the fact that DNA-DSBs are found at an equal frequency in rearranged and germline Vλ1 genes, the frequency of mutations differs. While 57% (42/74) of rearranged Vλ1 genes were mutated at a frequency of 0.61% (144 mutations in 23680 base pairs sequenced), only 21% (7/34) of germline Vλ1 genes sequenced were mutated at a frequency of 0.11% (12 mutations in 10880 base pairs sequenced). Taking into account that SHM has been active in 57% of the cells, the actual mutation frequency is 1.07% for rearranged and 0.19% for germline Vλ1 genes. In line with previous studies, about 60% of class switched $V\lambda1^{+}$, $CD19^{+}$, Igμδ memory B cells have a mutated, rearranged Vλ1 gene

(Fukita *et al.*, 1998; Jacobs *et al.*, 1998) and mutations in germline configured Vλ segments occur, albeit at a significantly lower frequency (Azuma, 1998). Therefore, although DNA-DSBs are introduced at similar frequencies in germline and rearranged Vλ1&2 genes, SHM is preferentially targeted to the rearranged Vλ1&2 genes, suggesting that a DNA-DSB itself does not suffice for optimal targeting of the hypermutation machinery. We propose that usually most DNA-DSBs are efficiently repaired in a non-mutagenic manner and therefore, do not lead to cell death. In the presence of AID, however, the likelyhood that the processing of the lesions (nicks or DNA-DSBs) becomes error prone increases, and the risk of disfavored mutations potentially leading to cell death or abberant development, like lymphomas and autoimmunity.

In conclusion, despite the fact that DNA-DSBs are found at similar frequencies in germline and rearranged Vλ gene segments, SHM is preferentially targeted to rearranged Vλ gene segments. As the generation of DNA-DSBs is dependent on transcription, or at least on the initiation of transcription, the occurrence of DNA-DSBs in germline and rearranged Vλ genes at a similar frequency likely relates to the presence of two independent λ enhancers. The data clearly show that most of the DNA-DSBs are AID independent. Therefore three possibilities have been raised (Bross *et al.*, 2002); (1) none of the DNA-DSBs are involved in SHM, (2) only a small fraction of DNA-DSBs are involved in SHM and (3) DNA-DSBs are generated in transcription dependent but AID independent manner. The latter possibility suggests that AID function was established to utilize these lesions in order to mediate Ig gene remodeling like CSR, SHM or gene conversion. Present studies aim on the identification of the nuclease and the identification of the AID substrate.

### MATERIAL AND METHODS

### Mice

The generation and genotyping of AID- and Igκ-deficient mice have been described elsewhere (Zou *et al.*, 1993; Muramatsu *et al.*, 2000).

### Immunization

Igκ K.O. mice were immunized with 0.2 ml of a 10% sheep red blood cells solution in PBS (Bross *et al.*, 2000). AID K.O. mice were immunized with NP-CG. For the immunization with NP-CG, NP(28)-CGG® (Biosearch Technologies Inc.) is resuspended at 1 mg/ml in PBS, an equal volume of Alu-Gel-S® (Serva) is added, mixed, incubated over night at 4°C, and 0.2 ml of this suspension (100 μg NP(28)-CGG) is injected i.p. For the analysis of DNA-DSBs mice were sacrificed 7 days after immunization, for the analysis of SHM, mice were sacrificed 10 days after immunization.

### Cell Sorting, and DNA Isolation

Sorting of GC and non-GC B cells was done using a combination of magnet activated cell sorting (MACS) (Miltenyi Biotec) and fluorescence activated cell sorting (FACS) using a FACStar™ (Becton Dickinson). The isolation of high molecular weight DNA from these fractions has been described elsewhere (Ref). Sorting of Vλ1&2 expressing GC and non-GC B cell subsets was achieved with a Vλ1&2 specific monoclonal antibody (clone LS136) as described in Jacobs *et al.* (1998).

### Analysis of SHM in Germline Vλ1 Gene Segments

For the amplification of germline Vλ1 gene segments a semi-nested PCR assay was applied using the reverse Vλ1 intron primer in combination with the Vλ1&2 external primer for the 1st round and the Vλ1&2 internal primer for the 2nd round of PCR amplification. The PCR amplification was performed as described (Jacobs *et al.*, 1998).

### Amplification, Cloning and Sequencing of Splinkerette-ligated Vλ Genes

The ligation of the splinkerettes has been described elsewhere (Jacobs *et al.*, 1998). The quantity of the DNA used is based on a defined number of sorted cells. Based on previous experiments and as determined by semi-quantitative PCR reactions with Ku70 specific primers (see below) this method of DNA quantification is reproducible. Specific amplification of adapter-ligated Vλ1&2 genes from genomic DNA was achieved by using a nested PCR strategy. In the first round, the external splinkerette primer was used in combination with the external Vλ primer. For the second round of amplification the internal splinkerette primer was used in combination with the internal Vλ primer. To detect any Vλ/adapter hybrids, we used the same PCR conditions as described for the amplification of the Vλ1 genes from single cells (Jacobs *et al.*, 1998). PCR products were resolved on a 2% (w/v) agarose gel, visualized with ethidium bromide under UV-light and isolated from agarose gel slices using the QiaQuick® matrix (Qiagen). After isolation the PCR products were cloned into the TOPO pCRII® vector from the TOPO TA Cloning® kit (Invitrogen) and sequenced using the DyeDeoxy Terminator Cycle Sequencing kit® (Applied Biosystems).

### Oligonucleotides

Vλ1&2 external primer: 5′-GGGTATGCAACAATGCG-CATCTTGTC-3′, Vλ1&2 internal primer: 5′-GCGAA-GCGAAGAGAAGCTTGTGACTCAGGAATCTGCA-3′,

Vλ1 intron primer 5′-AATGATTCTATGTTCTGCCAAGTC-3′. External splinkerette primer: 5′-CGAAGAGTAACCGTTGCTAGGAGAGACC-3′. Internal splinkerette primer: 5′-GTGGCTGAATGAGACTGGTGTCGAC-3′. Splinkerette oligomers: 5′CGAAGAGTAACCGTTGCTAGGAGAGACCGTGGCTGAATGAGACTGGTGTCGACACTAGTGG-3′ (long strand, 61-mer), 5′-CCACTAGTGTCGACACCAGTCTCTAATTTTTTTTTTCAAAAAAA-3′ (short strand, 44-mer), 5′ACACGGCTTCCTTAATGTGA-3′ (KU70 forward primer), and 5′ GGCTGGCTTTAGCACTGTCA (KU70 reverse primer).

### Acknowledgements

The authors like to thank Sue Cooper and Roy Allenspach for expert technical assistance, Tracy Hayden and Hubertus Kohler for fluorescence activated cell sortings, Erwin Schilliger for preparing figures, the BII animal caretaker team for their biotechnical help, and F. McBlane and G. Kline for critically reading the manuscript. Many thanks to Drs Sigfried Weiss and Holger Engel at the GBF in Braunschweig, Germany for providing valuable sequence information on the mouse Igλ locus and to F. Hoffmann La Roche Ltd, Basel, Switzerland, which founded and supported the Basel Institute for Immunology.

### References

Arakawa, H., Hauschild, J. and Buerstedde, J.M. (2002) "Requirement of the activation-induced deaminase (AID) gene for immunoglobulin gene conversion", *Science* **295**, 1301–1306.

Azuma, T. (1998) "Somatic hypermutation in mouse lambda chains", *Immunol. Rev.* **162**, 97–105.

Bemark, M., Sale, J.E., Kim, H.J., Berek, C., Cosgrove, R.A. and Neuberger, M.S. (2000) "Somatic hypermutation in the absence of DNA-dependent protein kinase catalytic subunit (DNA-PKcs) or recombination-activating gene (RAG)1 activity", *J. Exp. Med.* **192**, 1509–1514.

Bross, L., Fukita, Y., McBlane, F., Demolliere, C., Rajewsky, K. and Jacobs, H. (2000) "DNA double-strand breaks in immunoglobulin genes undergoing somatic hypermutation", *Immunity* **13**, 589–597.

Bross, L., Muramatsu, M., Kinoshita, K., Honjo, T. and Jacobs, H. (2002) "DNA double-strand breaks: prior to but not sufficient in targeting hypermutation", *J. Exp. Med.* **195**, 1187–1192.

Fukita, Y., Jacobs, H. and Rajewsky, K. (1998) "Somatic hypermutation in the heavy chain locus correlates with transcription", *Immunity* **9**, 105–114.

Goossens, T., Klein, U. and Kuppers, R. (1998) "Frequent occurrence of deletions and duplications during somatic hypermutation: implications for oncogene translocations and heavy chain disease", *Proc. Natl Acad. Sci. USA* **95**, 2463–2468.

Honjo, T., Kinoshita, K. and Muramatsu, M. (2002) "Molecular mechanism of class switch recombination: linkage with somatic hypermutation", *Annu. Rev. Immunol.* **20**, 165–196.

Jacobs, H. and Bross, L. (2001) "Towards an understanding of somatic hypermutation", *Curr. Opin. Immunol.* **13**, 208–218.

Jacobs, H., Fukita, Y., van der Horst, G.T., de Boer, J., Weeda, G., Essers, J., de Wind, N., Engelward, B.P., Samson, L., Verbeek, S., de Murcia, J.M., de Murcia, G., te Riele, H. and Rajewsky, K. (1998) "Hypermutation of immunoglobulin genes in memory B cells of DNA repair- deficient mice", *J. Exp. Med.* **187**, 1735–1743.

Jacobs, H., Puglisi, A., Rajewsky, K. and Fukita, Y. (1999) "Tuning somatic hypermutation by transcription", *Curr. Top. Microbiol. Immunol.* **246**, 149–158.

Kong, Q. and Maizels, N. (2001) "DNA breaks in hypermutating immunoglobulin genes: evidence for a break- and-repair pathway of somatic hypermutation", *Genetics* **158**, 369–378.

Lellek, H., Kirsten, R., Diehl, I., Apostel, F., Buck, F. and Greeve, J. (2000) "Purification and molecular cloning of a novel essential component of the apolipoprotein B mRNA editing enzyme-complex", *J. Biol. Chem.* **275**, 19848–19856.

Martin, A., Bardwell, P.D., Woo, C.J., Fan, M., Shulman, M.J. and Scharff, M.D. (2002) "Activation-induced cytidine deaminase turns on somatic hypermutation in hybridomas", *Nature* **415**, 802–806.

Mehta, A., Kinter, M.T., Sherman, N.E. and Driscoll, D.M. (2000) "Molecular cloning of apobec-1 complementation factor, a novel RNA- binding protein involved in the editing of apolipoprotein B mRNA", *Mol. Cell. Biol.* **20**, 1846–1854.

Muramatsu, M., Sankaranand, V.S., Anant, S., Sugai, M., Kinoshita, K., Davidson, N.O. and Honjo, T. (1999) "Specific expression of activation-induced cytidine deaminase (AID), a novel member of the RNA-editing deaminase family in germinal center B cells", *J. Biol. Chem.* **274**, 18470–18476.

Muramatsu, M., Kinoshita, K., Fagarasan, S., Yamada, S., Shinkai, Y. and Honjo, T. (2000) "Class switch recombination and hypermutation require activation-induced cytidine deaminase (AID), a potential RNA editing enzyme", *Cell* **102**, 553–563.

Nagaoka, H., Muramatsu, M., Yamamura, N., Kinoshita, K. and Honjo, T. (2002) "Activation-induced deaminase (AID)-directed hypermutation in the immunoglobulin Smu region: implication of AID involvement in a common step of class switch recombination and somatic hypermutation", *J. Exp. Med.* **195**, 529–534.

Neuberger, M.S. and Milstein, C. (1995) "Somatic hypermutation", *Curr. Opin. Immunol.* **7**, 248–254.

Okazaki, I.M., Kinoshita, K., Muramatsu, M., Yoshikawa, K. and Honjo, T. (2002) "The AID enzyme induces class switch recombination in fibroblasts", *Nature* **416**, 340–345.

Papavasiliou, F.N. and Schatz, D.G. (2000) "Cell-cycle-regulated DNA double-stranded breaks in somatic hypermutation of immunoglobulin genes", *Nature* **408**, 216–221.

Petersen, S., Casellas, R., Reina-San-Martin, B., Chen, H.T., Difilippantonio, M.J., Wilson, P.C., Hanitsch, L., Celeste, A., Muramatsu, M., Pilch, D.R., Redon, C., Ried, T., Bonner, W.M., Honjo, T., Nussenzweig, M.C. and Nussenzweig, A. (2001) "AID is required to initiate Nbs1/gamma-H2AX focus formation and mutations at sites of class switching", *Nature* **414**, 660–665.

Revy, P., Muto, T., Levy, Y., Geissmann, F., Plebani, A., Sanal, O., Catalan, N., Forveille, M., Dufourcq-Labelouse, R., Gennery, A., Tezcan, I., Ersoy, F., Kayserili, H., Ugazio, A.G., Brousse, N., Muramatsu, M., Notarangelo, L.D., Kinoshita, K., Honjo, T., Fischer, A. and Durandy, A. (2000) "Activation-induced cytidine deaminase (AID) deficiency causes the autosomal recessive form of the Hyper-IgM syndrome (HIGM2)", *Cell* **102**, 565–575.

Wijgerde, M., Grosveld, F. and Fraser, P. (1995) "Transcription complex stability and chromatin dynamics *in vivo*", *Nature* **377**, 209–213.

Zou, Y.R., Gu, H. and Rajewsky, K. (1993) "Generation of a mouse strain that produces immunoglobulin kappa chains with human constant regions", *Science* **262**, 1271–1274.

